# Toxic Evaluations of *Calea phyllolepis* Extracts Standardized on 6‐*epi*‐β‐Verbesinol Coumarate and Its In Silico Prediction of the Toxicity

**DOI:** 10.1002/cbdv.202501277

**Published:** 2025-09-25

**Authors:** Suele Bierhals Vencato, Guilherme Borsoi, Cleverson Feistel, Mariele Feiffer Charão, Angélica Rocha Joaquim, Jaqueline Nascimento Picada, Alexandre de Barros Falcão Ferraz

**Affiliations:** ^1^ Scientific Initiation Program Lutheran University of Brazil (ULBRA) Canoas Brazil; ^2^ Laboratory of Toxicological Analyses, Institute of Health Sciences Feevale University Novo Hamburgo Brazil; ^3^ Postgraduate Program in Pharmaceutical Sciences, Center of Health Sciences Federal University of Santa Maria (UFSM) Santa Maria Brazil; ^4^ Laboratory of Genetic Toxicology Lutheran University of Brazil (ULBRA) Canoas Brazil

**Keywords:** Ames test, Asteraceae, *Caenorhabditis elegans*, in silico, phenolic acid

## Abstract

Medicinal plants are traditionally used in folk medicine. Still, there is a misconception about the safety/efficacy of natural treatments, which results in few studies on the toxic, genotoxic, and mutagenic potential of plants. Therefore, this work investigates the toxicological and mutagenic potential of an ethanolic extract and fractions of *Calea phyllolepis* leaves using *Caenorhabditis elegans* and *Salmonella typhimurium* assays. Through the results obtained, it was verified that only the hexane fraction induced toxicity in *C. elegans*, affecting the survival and development of nematodes. In addition, this fraction was mutagenic through the *S. typhimurium* assay only in the presence of metabolization. A previous study pointed out that the major compound identified in the hexane fraction, 6‐*epi*‐β‐verbesinol coumarate, was responsible for the cytotoxic effects. Probably due to the fragility of the carbon–oxygen bond of the 6‐*epi*‐β‐verbesinol coumarate, this substance undergoes degradation, generating verbesinol (sesquiterpene) and coumaric acid (phenolic acid) in the body in vivo as active metabolites. Based on the in silico predictions for 6‐*epi*‐β‐verbesinol coumarate metabolism and toxicity, 21 metabolites and 7 potentially mutagenic products were identified, belonging to three classes: epoxides (three metabolites), α,β‐unsaturated carbonylated compounds (one metabolite), and simple aldehydes.

## Graphical abstract

In vivo and in silico toxicological effects of *Calea phyllolepis* extracts Standardized on 6‐epi‐β‐Verbesinol Coumarate.

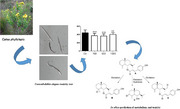

## 1 Introduction

The use of plant‐based medicines for treating diseases has significantly increased, attributed to their fewer side effects compared to synthetic drugs. However, many medicinal plants for primary medical care or as dietary supplements have not been scientifically evaluated, and their potential side effects need to be assessed. Therefore, it is essential to conduct toxicity studies to ensure the safety of these plants [1, 2].

The genus *Calea* is used in Latin American folk medicine to treat rheumatism, respiratory diseases, and digestive disorders [3]. However, many other widespread indications have been reported, as *Calea zacatechichi* is a plant used for ritual in Mexico [4, 5]. *Calea uniflora*, also known as “arnica,” is a native plant to the South of Brazil used to treat rheumatism, respiratory diseases, and digestive problems [6]. *Calea urticifolia* is used in the folkloric medicine of El Salvador for its calming effects on diarrhea and fever [2]. In addition to the activities indicated by popular use, we can find in the scientific literature other biological properties that reveal more bioactive profiles acting against distinct microorganisms, such as antimalarial [7], antileishmanial [8, 9], acaricidal [10], antimycobacterial [11], antihelminthic [12], antibacterial, and antifungal [13].

Despite these activities, toxicological studies are needed to ensure the safe and rational use of species from this genus. González‐Yáñez et al. [14] reported that in vitro, the extract of *Calea ternifolia* (*C. zacatechichi*) induced eryptosis and inhibited CYP3A. In vivo, the extract showed moderate toxicity to *A. salina*, reduced platelet and leukocyte counts, and increased levels of urea and liver enzymes, including alanine aminotransferase (ALT), aspartate aminotransferase (AST), and alkaline phosphatase (ALP). The cellular and mitochondrial functional changes in human proximal tubule HK‐2 cells indicated the toxicity of *C. ternifolia* (*C. zacatechichi*), and even at low doses, evidence of cellular toxicity was detected. Moreover, these findings correlated with significantly elevated levels of nephrotoxicity biomarkers, supporting the need to further scrutinize the safety of this herbal dietary supplement [15].

Many plant‐derived compounds show therapeutic potential; however, the assumption that natural products are safe is incorrect. Herbal preparations often contain compounds that can exert pharmacological or toxicological effects. Due to this variability, the toxicological evaluation of biologically active compounds and extracts is essential before pharmacological testing to ensure appropriate dosing and safe development of drugs [16–19].

Numerous species remain to be studied in the Pampa biome, located in southern Brazil, numerous species remain to be studied. The selection of *Calea* species is linked to the presence of taxonomic markers such as sesquiterpene lactones, benzofurans, and benzopyrans owing to their potent bioactivities, including cancer cell cytotoxicity and antineoplastic efficacy in in vivo studies. In our search for bioactive compounds from natural sources against cancer, *Calea phyllolepis*, a plant endemic to Brazil, was found to have hexane and ethyl acetate fractions that showed higher cytotoxic effects. The major bioactive compound from the hexane fraction, 6‐*epi*‐β‐verbesinol coumarate, exhibited low cytotoxicity to normal fibroblast cells, suggesting a high selectivity index (SI = 7.39) against breast cancer cells [20]. Based on this relevant antiproliferative data and the lack of toxicological studies on species from this genus, this study focused on assessing the toxicological potential of the ethanolic extract and fractions from *C. phyllolepis* leaves using *Caenorhabditis elegans* and *Salmonella*/microsome tests.

## 2 Materials and Methods

### 2.1 Plant Material

The leaves of *C. phyllolepis* Baker were collected and identified by Dr. Sérgio Bordignon in April 2018 at Santo Antônio da Patrulha, RS—Brazil (29°50′18″ S, 50°30′58″ W). The botanical material was deposited at the Herbarium of the Universidade Federal do Rio Grande do Sul (ICN 199206). Access to Brazilian biodiversity was registered in the National System of Genetic Resource Management and Associated Traditional Knowledge (SISGEN) under a protocol (AEFB71B).

### 2.2 Chemicals

Dimethyl sulfoxide, quercetin, gallic acid, 2‐aminoanthracene, 4‐nitroquinoline oxide (4‐NQO), Folin–Ciocalteu's phenol reagent, and all solvents were obtained from Sigma‐Aldrich (Darmstadt, Germany).

### 2.3 Crude Ethanolic Extract

Fifteen grams of *C. phyllolepis* leaves were extracted in a Soxhlet apparatus with ethanol. After 4 h of extraction, the extract was evaporated to dryness under reduced pressure (<50°C), yielding the ethanolic extract (1.83 g—12.2%)

### 2.4 Preparation of the Fractions

Thirty grams of *C. phyllolepis* leaves were extracted successively in a Soxhlet apparatus. The fractionation followed the solvents in increasing order of polarity (hexane, ethyl acetate, and methanol). After 4 h of extraction, each fraction was evaporated to dryness under reduced pressure (<50°C), yielding the hexane fraction (1.74 g—5.8%), ethyl acetate fraction (3.15 g—10.5%), and methanolic fraction (7.92 g—26.4%).

### 2.5 Total Phenolic Compounds Content

The total phenolic compound content of ethanolic extract from *C. phyllolepis* leaves was carried out in triplicate and followed by the Folin–Ciocalteu method. The amount was expressed as gallic acid equivalent (GAE) in mg/g extract [21]. The absorption was read at 765 nm in the Shimadzu spectrophotometer (UV–1602PC, Kyoto, Japan).

### 2.6 Total Flavonoid Content

The total flavonoid content of ethanolic extract from *C. phyllolepis* leaves was determined by a colorimetric method based on the formation of stable complexes between the aluminum cation and flavonoids present in the sample as described by Woisky and Salatino [22]. The analyses were carried out in triplicate using a Shimadzu spectrophotometer (UV–1602PC, Kyoto, Japan), and the total flavonoid content in the samples was expressed as quercetin equivalents (QE) in mg/g of extract.

### 2.7 Gas Chromatography–Mass Spectroscopy Analysis

The hexane, ethyl acetate, and methanolic fractions and the ethanolic extract of *C. phyllolepis* were submitted to a gas chromatographic analysis performed using a Varian 450‐CG (Agilent Technologies, Santa Clara, CA, USA). The 6‐*epi*‐β‐verbesinol coumarate (98.10%) previously isolated was used to standardize the fractions through gas chromatography/mass spectrometry (GC/MS) analysis. All the data concerning isolation procedures and structure elucidation were described by da Rosa et al. (2022) [20]. The data were processed and analyzed using the MS Workstation software version 6.9.3 (Build November 30, 2008) produced by the company Varian. Chromatographic separation was achieved on an Agilent 19091S‐433HP‐5MS column (30 m × 0.25 mm i.d., film thickness 0.25 µm) with 5% phenylmethylsiloxane (HP‐5 MS), supplied by J&W Scientific (Folsom, CA, USA). GC/MS analysis was performed, using a temperature program at 50°C–325°C (30°C/min), flow rate 1.0 mL/min with helium 6.0 as the carrier gas, and a run time of 40.00 min. The injector temperature was maintained at 260°C in 1:10 split mode. The detector model ION TRAP was programmed to acquire ions in Full Scan mode with an analytical range of 20–600 *m*/*z*.

The electron ionization (EI) mass spectrometer was programmed in full scan mode with incident ionization of 70 eV. The instrumental components were submitted to internal calibration checks to ensure the accurate performance of the mass spectrometry. The limits of detection and quantification data were obtained through the calibration curve (*R* = 0.9870) and monitoring of the selected ions. All data were properly stored for checking, according to the quality assurance (QA) procedure.

### 2.8 *C. elegans* Toxicity Test

The toxic effect in *C. elegans* was evaluated using the N2 wild‐type strain obtained from the *Caenorhabditis* Genetics Center (CGC) (University of Minnesota, Twin Cities, MN, USA) as described in previous studies [23, 24]. The strain was maintained at 20°C in a nematode growth medium (NGM) with *Escherichia coli* OP50 as a food source [25]. Before the experiments, the nematodes were synchronized to obtain the L1 larval stage. Briefly, the gravid nematodes were removed from the plate through sustained washes and were exposed to a bleaching solution (1% NaOCl; 0.25 M NaOH) to rupture the nematode's cuticle, and the eggs were collected by flotation using a sucrose solution (30%, m/v) and were placed on an NGM plate without bacteria. After 12–14 h of incubation, the L1 stage of the nematodes was obtained.

A total of 1500 synchronized L1 worms per concentration were used in a liquid medium containing the M9 buffer (0.02 M KH_2_PO_4_, 0.04 M Na_2_HPO_4_, 0.08 M NaCl, and 0.001 M MgSO_4_). The nematodes were treated with 2 mL of each 100, 500, and 1000 µg/mL of hexane, ethyl acetate, and methanolic fractions and ethanolic extract of *C. phyllolepis* in a 5‐mL polypropylene tube supplemented with *E. coli* OP50 (final concentration OD_600 nm_ = 1). These concentrations were based on the previous study, which showed inhibitory and bactericidal action for the ethanolic extract and hexane fraction of *C. phyllolepis* at 500 µg/mL (data not shown). The tubes were maintained under constant agitation in a rotator for 30 min at 20°C (acute exposition). After that, the nematodes were laid out on NGM plates seeded with *E. coli* OP50. The control group was treated only with M9 buffer, and it was used as 100% of the survival rate [25, 26]. After 24 h, the number of surviving worms on each plate was counted in a stereomicroscope.

The development was evaluated through the measurement of the body length of the nematodes as a toxic endpoint, according to previous studies [27, 28]. The treated nematodes of each group were placed on NGM plates, and after 48 h, the body lengths of the nematodes were evaluated (in the adult stage). For this procedure, 15 µL of the solution with the worms was mounted on 2% agarose pads with 15 µL of levamisole (22.5 mg/mL) to anesthetize.

The body length was measured by sampling. Twenty nematodes/treatments were photographed using a stereomicroscope coupled with a camera (TrueChrome Metrics, Precision), and their body lengths were measured using ImageJ software. All the experiments were performed in triplicate.

### 2.9 *Salmonella*/Microsome Mutagenicity Assay

The mutagenicity assay of the hexane fraction and the ethanolic extract was performed using *Salmonella typhimurium* strains (TA98 and TA100) according to the published method described in Mortelmans and Zeiger [29]. *S. typhimurium* strains were purchased from MOLTOX (Molecular Toxicology Inc., USA). Briefly, bacterial cultures were incubated at 37°C with different amounts of hexane fraction or ethanolic extract (250–5000 mg/plate) for 20 min without shaking. 2‐Aminoanthracene (2 µg/plate) was used as a positive control for all strains in the presence of metabolic activation (with S9 mix). In the mutagenic assays without S9 mix, sodium azide (1 µg/plate) was used as a positive control for the TA100 strain, and 4‐NQO (0.5 µg/plate) for the TA98. Plates were incubated in the dark at 37°C for 48 h before counting revertant colonies. Assays were repeated twice, and plating for each concentration was in triplicate.

### 2.10 In Silico Prediction of Metabolism and Toxicity of Metabolites

The software QSAR Toolbox 4.7 [30] was used to predict metabolites of the 6‐*epi*‐β‐verbesinol coumarate. The in vivo rat metabolism simulator was applied for predicting mutagenicity.

### 2.11 Statistical Analysis

The data on the survival and development of nematodes were evaluated by one‐way analysis of variance (ANOVA) followed by Bonferroni's test. The results from *Salmonella*/microsome assay were expressed as mean ± standard deviation (SD), and statistical significance was determined by ANOVA complemented by Dunnett's test for multiple comparisons, employing GraphPad Prism 5.0 software (GraphPad Inc., San Diego, CA, USA). Values of *p* < 0.05 were used to determine differences between groups. A test substance was considered mutagenic in the *Salmonella*/microsome assay when significant ANOVA variance was observed; the mean number of revertants on the test plates was at least twice as high as that observed in the negative control plates.

## 3 Results and Discussion

The total phenolic and flavonoid contents of the ethanolic extract of *C. phyllolepis* were analyzed. The results indicated a total phenolic content of 246.60 ± 3.79 mg/g EAG and a flavonoid content of 4.49 ± 0.07 mg/g EQ. In the literature, analyses of leaves from different plant species reported similar total phenolic contents: *Portulaca oleracea* had 216.96 mg/g, *Vernonia cinerea* showed 174.44 mg/g, and *Psidium guajava* had 256.76 mg/g of total phenolic content. Also, the total flavonoid amounts were found to be 6.07 mg/g in *Hovenia dulcis*, 6.97 mg/g in *Basella alba*, and 3.46 mg/g in *Aloysia gratissima* [31–35]. In addition, the content of 6‐*epi*‐β‐verbesinol coumarate was determined in the fractions and ethanolic extract using GC/MS. A higher concentration of 6‐*epi*‐β‐verbesinol coumarate was found in the hexane fraction (12.15%), followed by the ethanolic extract (7.23%) and acetate fraction (0.83%), whereas it was not detected in the methanolic fraction (see Data S1–S5).

**FIGURE 1:**
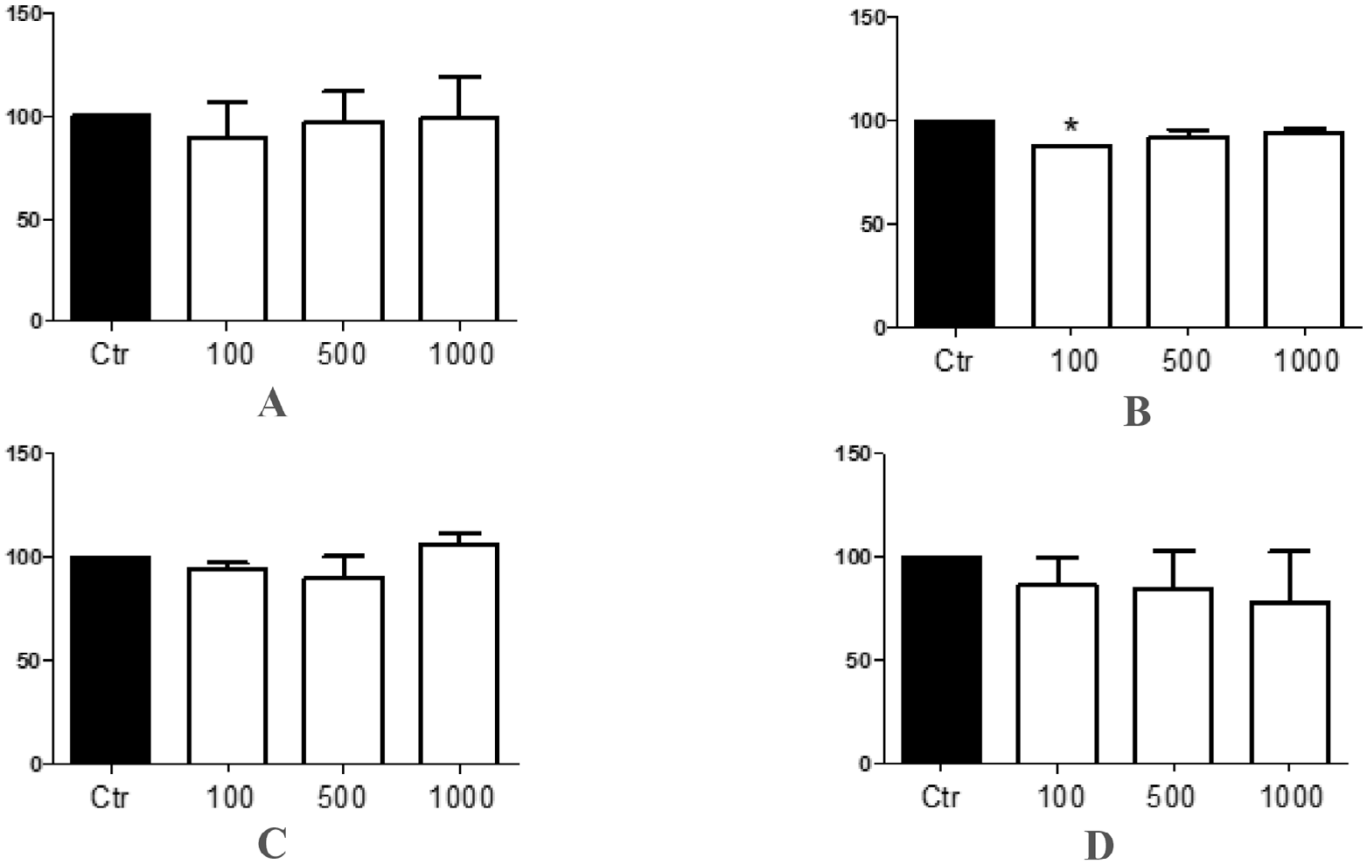
Survival of *Caenorhabditis elegans* exposed to different concentrations of *Calea phyllolepis* fractions and ethanolic extract. Ethanolic extract (A), hexanic fraction (B), ethyl acetate fraction (C), and methanolic fraction (D). Results *significant from control; *p < 0.05. ANOVA followed by Bonferroni's test.

**FIGURE 2:**
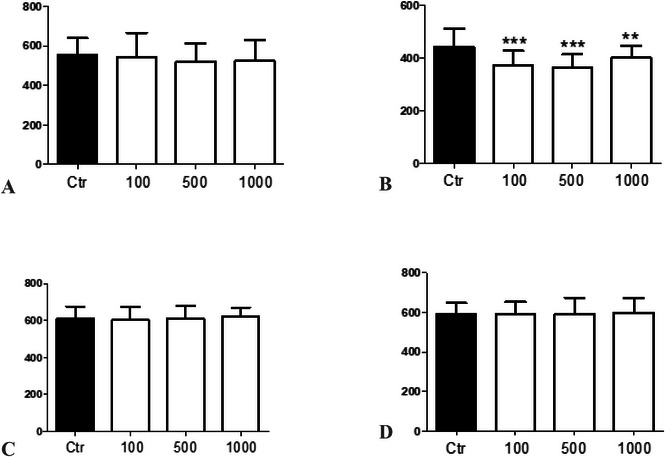
Development of *Caenorhabditis elegans* exposed to different concentrations of *Calea phyllolepis* fractions and ethanolic extract. Ethanolic extract (A), hexanic fraction (B), ethyl acetate fraction (C), and methanolic fraction (D). ****p < 0.0001 compared to control group; *p < 0.001 between concentration 500 and 1000 µg/mL. ANOVA followed by Bonferroni's test.

In our in vivo toxicological studies with *C. elegans*, we found that the ethanolic extract did not present toxicity in terms of survival rate (*F*(3, 8) = 0.3412, *p* = 0.7964), Figure 1A) and body length of nematodes (*F*(3, 156) = 0.4718, *p* = 0.7024, Figure 2A) as well as the ethyl acetate fraction (*F*(3, 8) = 2.737, *p* = 0.1776, Figure 1C; *F*(3, 156) = 0.8693, *p* = 0.4584, Figure 2C) and the methanolic fraction (*F*(3, 8) = 0.5986, *p* = 0.6490, Figure 1D; *F*(3, 156) = 0.1358, *p* = 0.9386, Figure 2D). Moreover, only the hexane fraction at the lowest concentration tested significantly decreased the survival rate of nematodes (*F*(3, 8) = 12.52, *p* = 0.0168, Figure 1B) compared to the control group and presented a significant reduction in the body length of nematodes in the 100 and 500 µg/mL groups compared to the control group and between the concentrations of 500 and 1000 µg/mL (*F*(3, 156) = 11.74, *p* < 0.0001, Figure 2B). In this study, it was not possible to determine the lethal dose (LD_50_) because 50% of the nematodes did not die, indicating the low toxicity of the extract and fractions.

The use of *C. elegans* as a toxicity model is widely recognized in the literature because of its advantages, such as ease of handling, low cost, and biological relevance [36, 37]. However, it is important to acknowledge its limitations, particularly the lack of metabolic activation enzymes, which are crucial for the bioactivation or detoxification of certain compounds [38]. This absence may lead to an underestimation or overestimation of toxic effects, depending on the role of metabolism in the activation or detoxification process of the tested agents. Therefore, while the model is useful for preliminary toxicity assessments, the results should be interpreted with caution, considering this limitation [36, 39]. Discussing these constraints is essential to contextualize the findings and guide future validations in models with enhanced metabolic capacity, such as rodent or human cell systems with comprehensive enzymatic activity [40].

In *C. elegans*, the absence of CYP1 orthologs may result in a reduced capacity to bioactivate coumarins, potentially limiting the generation of reactive intermediates compared to the rodent microsomal *S9* system. The genome of *C. elegans* encodes 75 CYP genes that are grouped into 16 different families [41]. Although some of these genes share homology with human CYP clans 2, 3, and 4, none are related to the CYP1 family [42]. However, the hexane fraction exhibited toxic effects, impairing somatic development in *C. elegans*, likely due to the presence of 6‐*epi*‐β‐verbesinol coumarate and its metabolites.

Given the absence of studies on the toxicity of *C. phyllolepis*, we searched for data from studies on other species within the genus. Torres‐Rodriguez et al. [43] reported that the aqueous and ethanolic extracts of *C. urticifolia* leaves did not demonstrate acute toxicity in Wistar rats. In addition, da Rosa et al. [3] showed that both the aqueous extract and methanolic fraction of *C. phyllolepis* showed no cytotoxicity against the L‐929 mouse fibroblast line, which supports the lack of toxicity of the methanolic fraction in the *C. elegans* assay.

Regarding the mutagenicity results of the *Salmonella*/microsome assay, the ethanolic extract and the hexane fraction did not show mutagenic activity in the group without metabolic activation (Table 1). The ethanolic extract and hexane fraction significantly increased the number of revertant colonies only at 5000 µg/plate, but the MI was less than 2 in TA98 and TA100 strains. However, the hexane fraction showed mutagenic activity in the TA100 strain when metabolized with S9 mix (Table 2), but not in the TA98 strain, indicating that it induced base pair substitution mutations after metabolization. Similarly, the ethanolic extract did not induce mutations in either strain.

**TABLE 1:** Induction of *his*+ revertant colonies by ethanolic extract and hexane fraction of *Calea phyllolepis* in *Salmonella typhimurium* strains without metabolic activation.

Substance	Concentration (µg/plate)	TA98		TA100	
		Rev/plate^a^	MI^b^	Rev/plate^a^	MI^b^
NC^c^	—	20.7 ± 5.0		107.3 ± 16.3	—
Ethanolic extract	500	21.3 ± 2.3	1.03	123.5 ± 0.7	1.15
	1000	25.0 ± 6.2	1.21	135.0 ± 14.7	1.26
	2000	27.0 ± 4.2	1.30	138.0 ± 3.6	1.29
	5000	33.3 ± 5.1*	1.61	153.7 ± 25.0	1.43
PC^d^	0.5 (4NQO)	90.5 ± 7.8***	4.37	—	—
	1 (NaN_3_)	—	—	595.5 ± 40.3***	5.55
NC^c^	—	23.0 ± 5.0	—	190.3 ± 10.3	—
Hexane fraction	500	21.0 ± 3.6	0.91	178.3 ± 38.2	0.94
	1000	24.3 ± 1.5	1.06	197.7 ± 10.2	1.04
	2000	19.7 ± 4.0	0.86	253.7 ± 30.9	1.33
	5000	14.3 ± 5.5	0.62	351.0 ± 47.2**	1.84
PC^d^	0.5 (4NQO)	206.5 ± 0.7***	8.98	—	—
	1 (NaN_3_)			702.0 ± 94.9***	3.69

*Note*: Significant from NC: *p < 0.05, **p < 0.01, and ***p < 0.001 (ANOVA, Dunnett's test).

^a^ Number of revertant colonies/plate given as mean ± SD.

^b^ MI: mutagenic index (number of *his*+ colonies induced in the sample/number of spontaneous *his*+ colonies in the negative control).

^c^ NC: negative control (distilled water to ethanolic extract or DMSO 50% to hexane fraction).

^d^ PC: positive control: NaN_3_ (sodium azide) to TA100; 4‐NQO (4‐nitroquinoline oxide) to TA98.

**TABLE 2:** Induction of *his*+ revertant colonies by ethanolic extract and hexane fraction of *Calea phyllolepis* in *Salmonella typhimurium* strains with metabolic activation (S9 mix).

Substance	Concentration (µg/plate)	TA98		TA100	
		Rev/plate^a^	MI^b^	Rev/plate^a^	MI^b^
NC^c^	—	18.0 ± 2.9	—	99.7 ± 12.7	—
Ethanolic extract	500	12.8 ± 3.2	0.71	88.7 ± 29.5	0.89
	1000	15.0 ± 4.3	0.83	82.3 ± 10.5	0.83
	2000	17.7 ± 1.5	0.98	93.7 ± 10.1	0.94
	5000	25.0 ± 5.3	1.39	128.0 ± 9.5	1.28
PC^d^	2	90.0 ± 9.5***	5.00	293.5 ± 92.6***	2.94
NC^c^	—	18.0 ± 3.6	—	111.8 ± 24.0	—
Hexane fraction	500	19.3 ± 2.9	1.07	140.7 ± 20.7	1.26
	1000	21.0 ± 13.5	1.17	204.8 ± 67.2*	1.83
	2000	26.0 ± 4.4	1.44	292.8 ± 61.8***	2.62
	5000	29.3 ± 11.9	1.63	282.2 ± 40.2***	2.52
PC^d^	2	85.0 ± 5.7***	4.72	343.5 ± 21.9***	3.07

*Note*: Significant from NC: *p < 0.05 and **p < 0.001 (ANOVA, Dunnett's test).

^a^ Number of revertant colonies/plate given as mean ± SD.

^b^ MI: mutagenic index (number of *his*+ colonies induced in the sample/number of spontaneous *his*+ colonies in the negative control).

^c^ NC: negative control (distilled water to ethanolic extract or DMSO 50% to hexane fraction).

^d^ PC: positive control: 2‐aminoanthracene.

The toxicity of the hexane fraction is supported by the chemical composition of the *Calea* genus, which contains a wide variety of phytochemical classes, including sesquiterpene lactones, benzofurans, benzopyrans, chromones, acetophenones, flavonoids, and chalcones [44]. Sesquiterpene lactones are an important class of phytochemical products responsible for the antiproliferative, cytotoxic [45], and antitumor [46] activities of the *Calea* species. In addition, cytotoxic effects have been reported for monoterpenes [47], sesquiterpenes [48], and benzopyrans [49, 50].

**FIGURE 3:**
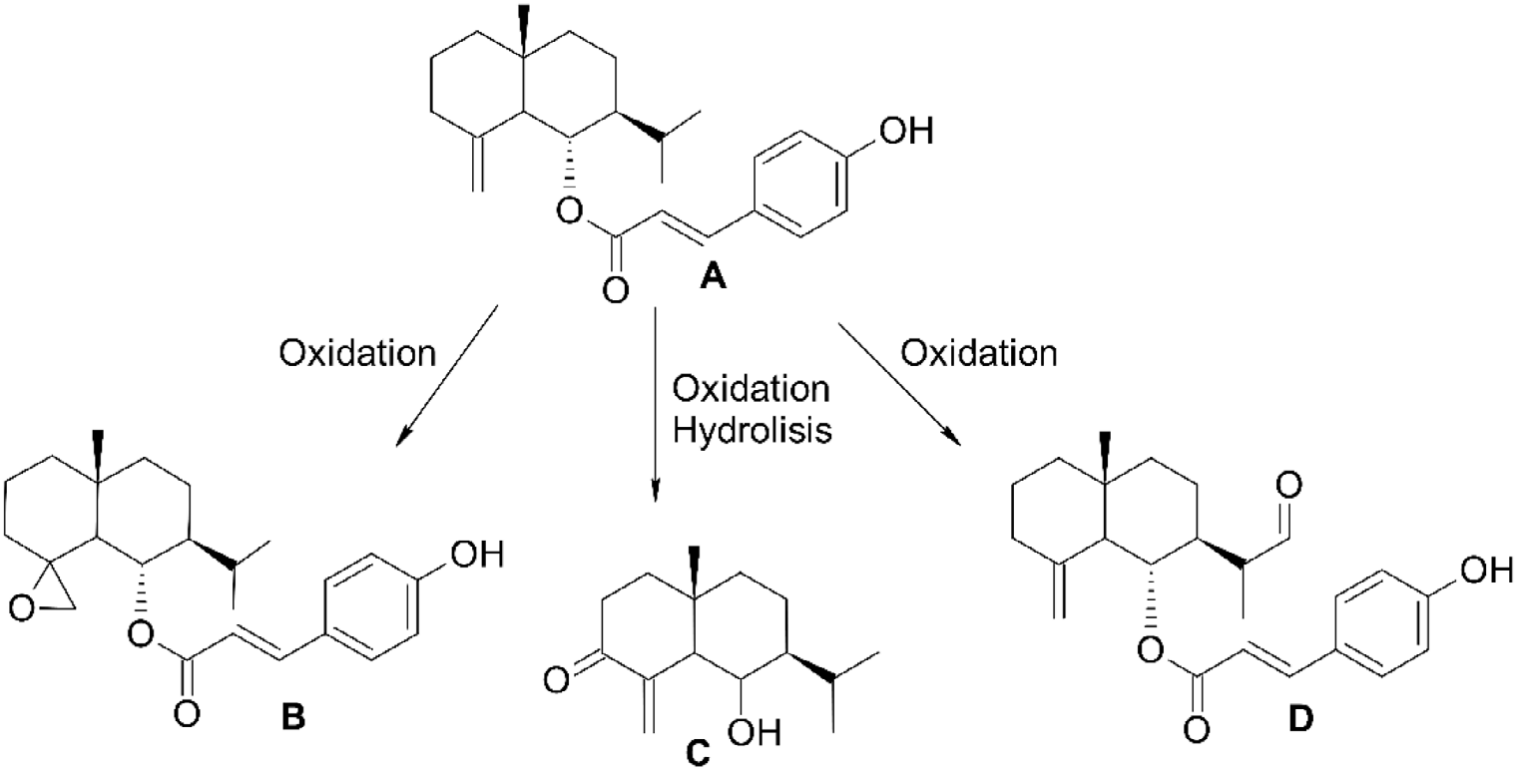
Chemical structure of 6‐*epi*‐β‐verbesinol coumarate (A) and one representative metabolite of each identified class in the in silico study: epoxide (B), α,β‐unsaturated carbonyl compound (C), and simple aldehyde (D).

Interestingly, the mutagenic effect of the hexane fraction only occurred after metabolization in the *S. thyphymurium* TA100 strain, which detects base‐pair substitution mutations. This suggests that the compounds present in the hexane fraction were biotransformed by microsomal enzymes, such as P450 in S9mix, leading to the production of metabolites with toxicological and mutagenic potential. A study by da Rosa et al. [20] identified 6‐*epi*‐β‐verbesinol coumarate (Figure 3A) in the leaves of *C. phyllolepis*. This compound was found to have the highest concentration in the hexane fraction (12.5%), followed by the ethanolic extract (7.23%) among all samples tested, which might have contributed to these findings. The P450 enzymes present in the *S9* mix can metabolize coumarins, converting them into epoxidized and/or hydroxylated metabolites, mainly by CYP1A, CYP2E1, and CYP2A6 [51, 52]. A review by Lake [53] revealed that coumarins induced mutations in the TA100 strain, but only in the presence of the *S9* mix.

The structure of this product consists of verbesinol (eudesmane‐type sesquiterpene) [54] linked via an oxygen atom to coumaric acid (phenolic acid). The toxic effects of the hexane fraction occur in metabolizing organisms, suggesting that the fragility of the ester present in 6‐*epi*‐β‐verbesinol coumarate leads to degradation, producing verbesinol and coumaric acid in vivo. Schar et al. [55] support these data, reporting the enzymatic hydrolysis of octadecyl ferulate in their study. In this study, the authors verified the disruption of the ester bond that joins ferulic acid (phenolic acid) to the octadecyl group, similar to the bond found in 6‐*epi*‐β‐verbesinol coumarate.

In a study by Porto et al. [56] on the toxic effects of phenolic acids in vivo, researchers found that gallic acid did not induce DNA damage when evaluated by the comet assay and micronucleus test at doses ranging from 10 to 100 mg/kg. Similarly, de Oliveira et al. [57] described the protective action of rosmarinic acid against ethanol‐induced genotoxic effects in rats, and also demonstrated the absence of genotoxicity and mutagenicity of this phenolic acid at a dose of 100 mg/kg.

On the other hand, eudesmane‐type sesquiterpenes have been found to exhibit cytotoxic activity in various plants. For example, these kinds of compounds were responsible for the cytotoxicity detected in the *Crepis sancta* and *Inula wissmanniana* extracts [58, 59]. In addition, eudesmane‐type compounds isolated from *Lindera glauca* have exhibited significant cytotoxicity against melanoma and colon carcinoma cell lines [60]. Extracts from *C. fruticosa* leaves have also demonstrated cytotoxic activity against colorectal carcinoma, ovarian, and glioblastoma cell lines. The most potent results were observed with the hexane fraction from which junenol was isolated [61, 44]. Studies have shown that (+)‐junenol and 6‐*epi*‐β‐verbesinol are the same compounds [62]. da Rosa et al. [3] corroborated these results, as the hexane fraction of *C. phyllolepis* leaves was again evidenced as the most active, and this effect was linked to the presence of the majority constituent in this fraction, since 6‐*epi*‐β‐verbesinol coumarate was evaluated alone and showed a more pronounced antiproliferative activity in all strains tested.

Finally, in silico analysis was performed to obtain more insights into the mutagenic potential of the hexane fraction. The metabolic profile and toxicity of 6‐*epi*‐β‐verbesinol coumarate metabolites were predicted using the QSAR Toolbox 4.7 software [30]. A total of 21 metabolites were identified, including verbesinol and *p*‐coumaric acid. Seven potentially mutagenic products were identified, belonging to three classes: epoxides (three metabolites), α,β‐unsaturated carbonylated compounds (one metabolite), and simple aldehydes (three metabolites) (Figure 3B–D). Some enzymatic metabolic transformations that generate these functional groups have been proposed in previous studies [63, 64]. Interestingly, epoxides, α,β‐unsaturated carbonylated molecules, and simple aldehydes have been identified as mutagenic compounds in the *Salmonella* model [65–67]. These metabolites possess an electrophilic center and can form covalent bonds with nucleophilic targets, which explains their mutagenic potential [68]. Epoxides are well‐known alkylating agents that are susceptible to nucleophilic attack by guanine in DNA [69, 70]. α,β‐Unsaturated carbonylated compounds can act as Michael acceptors and form adducts with DNA through covalent bonds. Aldehydes have also been identified as alkylating agents through adduct formation [71]. Taken together, the results of the in silico prediction and in vitro and in vivo toxicity evaluations suggest that the mutagenic effect of the hexane fraction can be associated with hydrolysis products and/or electrophilic metabolites.

## 4 Conclusions

The ethanolic extract and polar fractions did not exhibit toxicity in the *C. elegans* assay. However, the hexane fraction from *C. phyllolepis* exhibited toxic effects on the survival and development of *C. elegans* and mutagenic activity in the *Salmonella*/microsome assay. Notably, the harmful effects of the hexane fraction were only observed in the mutagenicity assay with metabolic activation (S9 mix). These results may be partially linked to the major constituent, 6‐*epi*‐β‐verbesinol coumarate, and its degradation compounds, which belong to three classes: epoxides, α,β‐unsaturated carbonylated compounds, and simple aldehydes, all of which are potentially mutagenic.

## Author Contributions

**Suele Bierhals Vencato**: writing – original draft, methodology, investigation. **Guilherme Bosoi**: writing – original draft, methodology. **Cleverson Feistel**: methodology, investigation. **Mariele Feiffer Charão**: methodology, investigation. **Angélica Rocha Joaquim**: writing – review and editing, software, conceptualization. **Jaqueline Nascimento Picada**: writing – review and editing, writing – original draft, validation, methodology, conceptualization. **Alexandre de Barros Falcão Ferraz**: writing – review and editing, writing – original draft, validation, methodology, conceptualization.

## Conflicts of Interest

The authors declare no conflicts of interest.

## Supporting information




**Supporting File 1**: cbdv70509‐sup‐0001‐SuppMat.pdf

## Data Availability

The data that support the findings of this study are available from the corresponding author upon reasonable request.
