# Supplementary material for: Toxic Evaluations of Calea phyllolepis Extracts Standardized on 6‐epi‐β‐Verbesinol Coumarate and Its In Silico Prediction of the Toxicity
Source: Chem Biodivers. 2025 Sep 25;22(12):e01277. doi: 10.1002/cbdv.202501277 (PMC12716012; doi:10.1002/cbdv.202501277)
Supplement: Supplementary file 1 — Supporting File 1: cbdv70509‐sup‐0001‐SuppMat.pdf [file CBDV-22-e01277-s001.pdf]

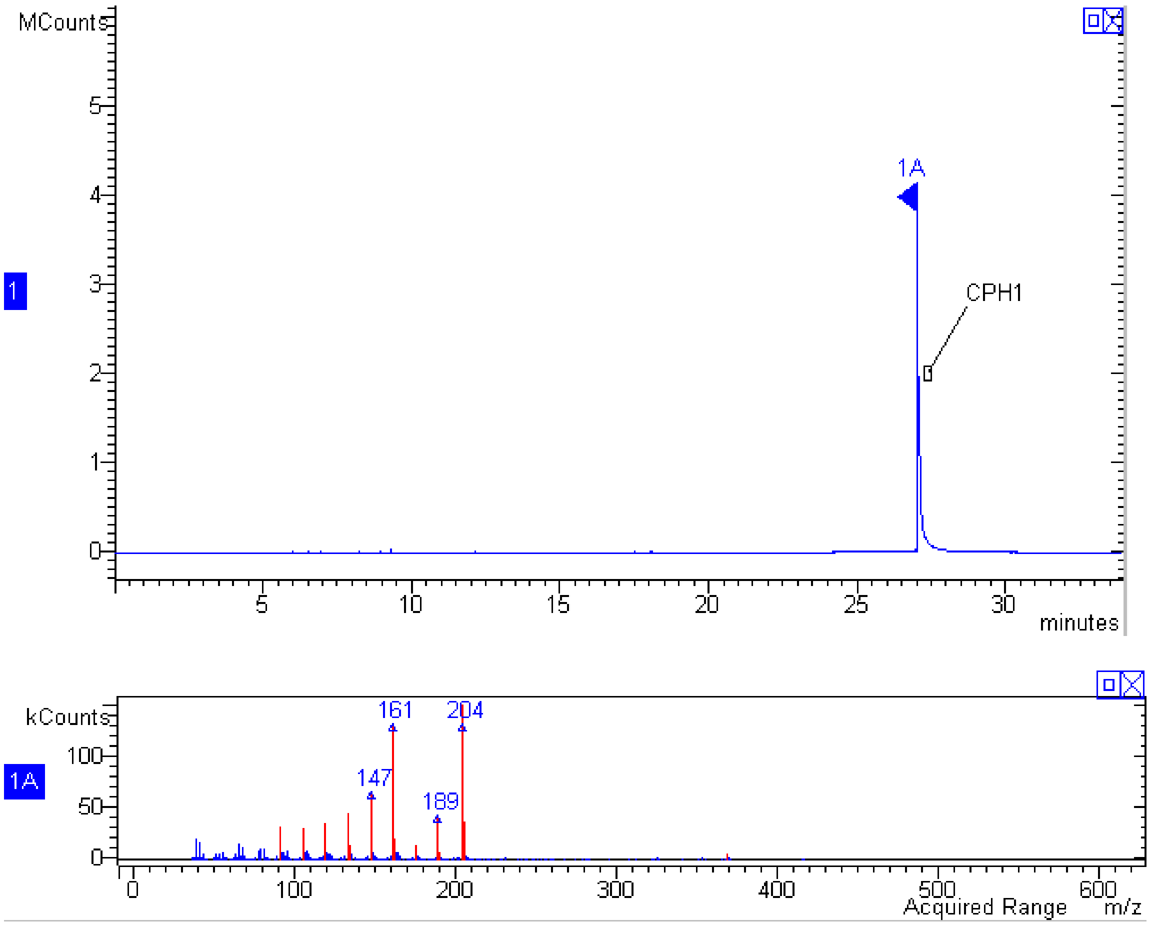

Supplementary data 1: Mass spectra and retention time of 6-epi-ß-verbesinol cumarate

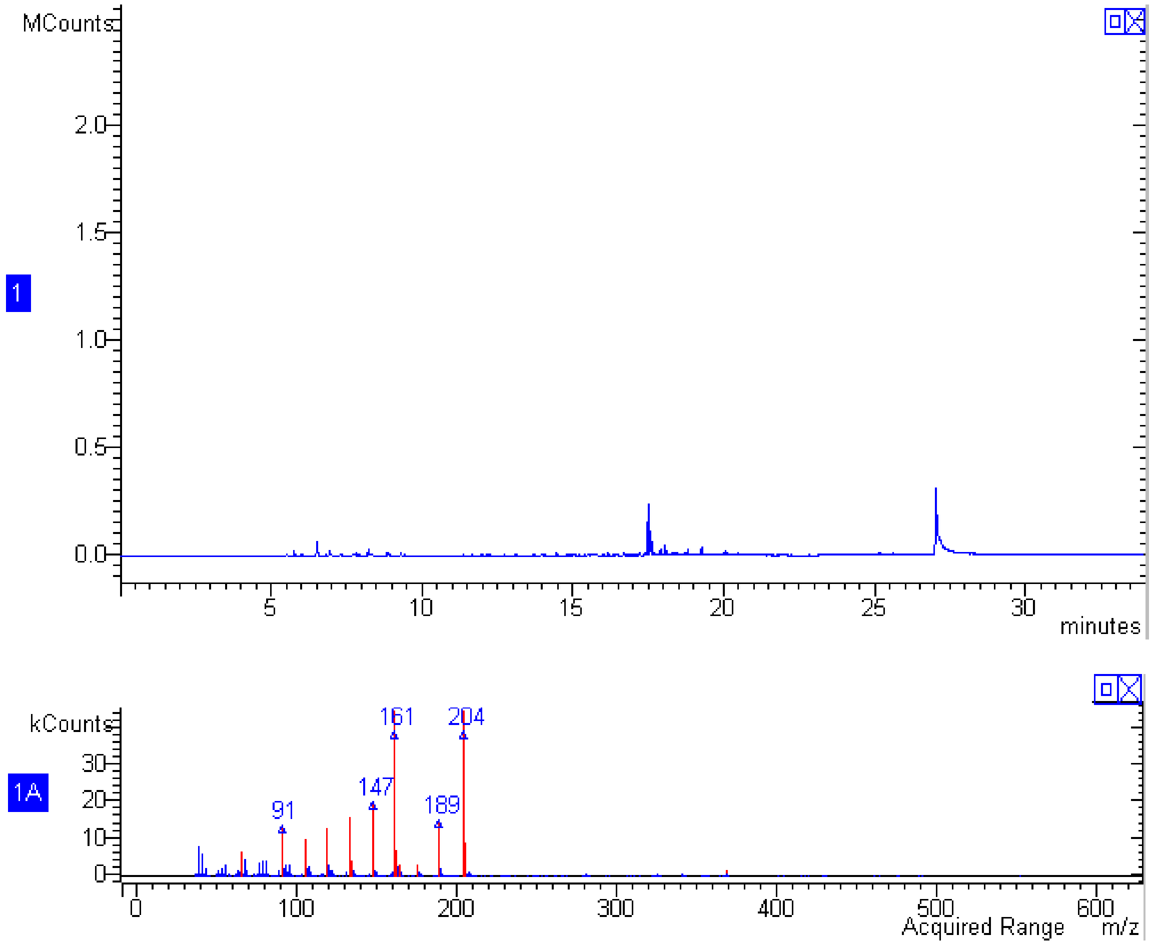

Supplementary data 2: Mass spectra and retention time of 6-epi-ß-verbesinol cumarate in the hexane fraction

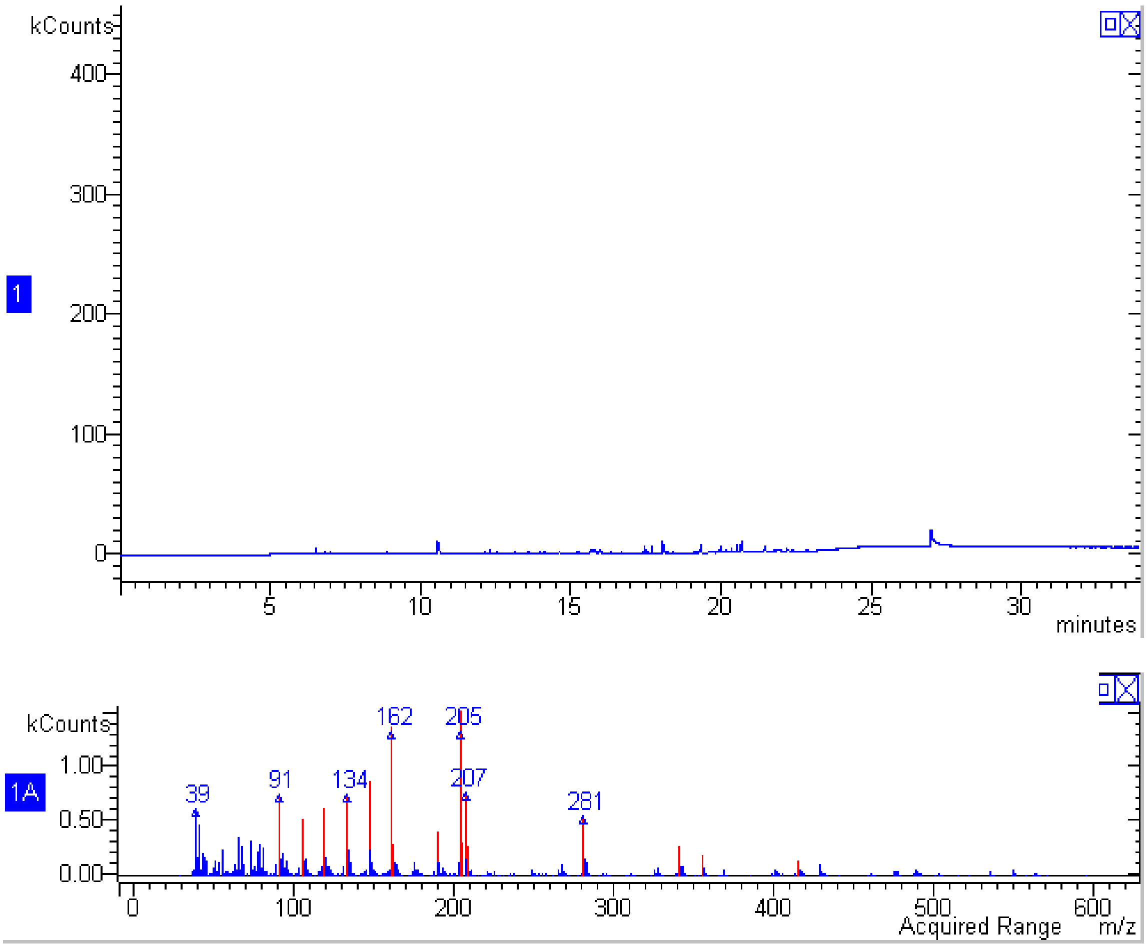

Supplementary data 3: Mass spectra and retention time of 6-epi-ß-verbesinol cumarate in the ethyl acetate fraction

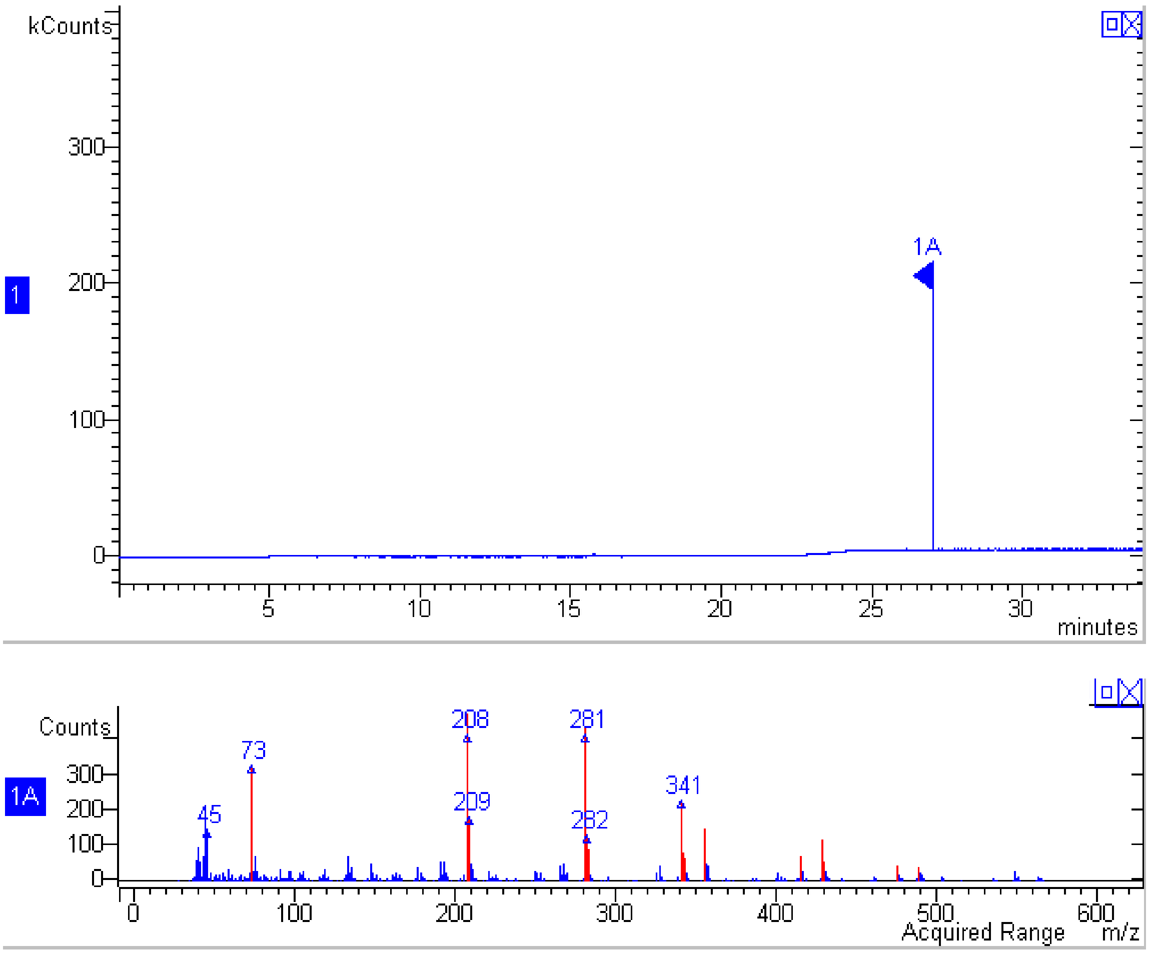

Supplementary data 4: Mass spectra and retention time of 6-epi-ß-verbesinol cumarate in the Metanolic fraction

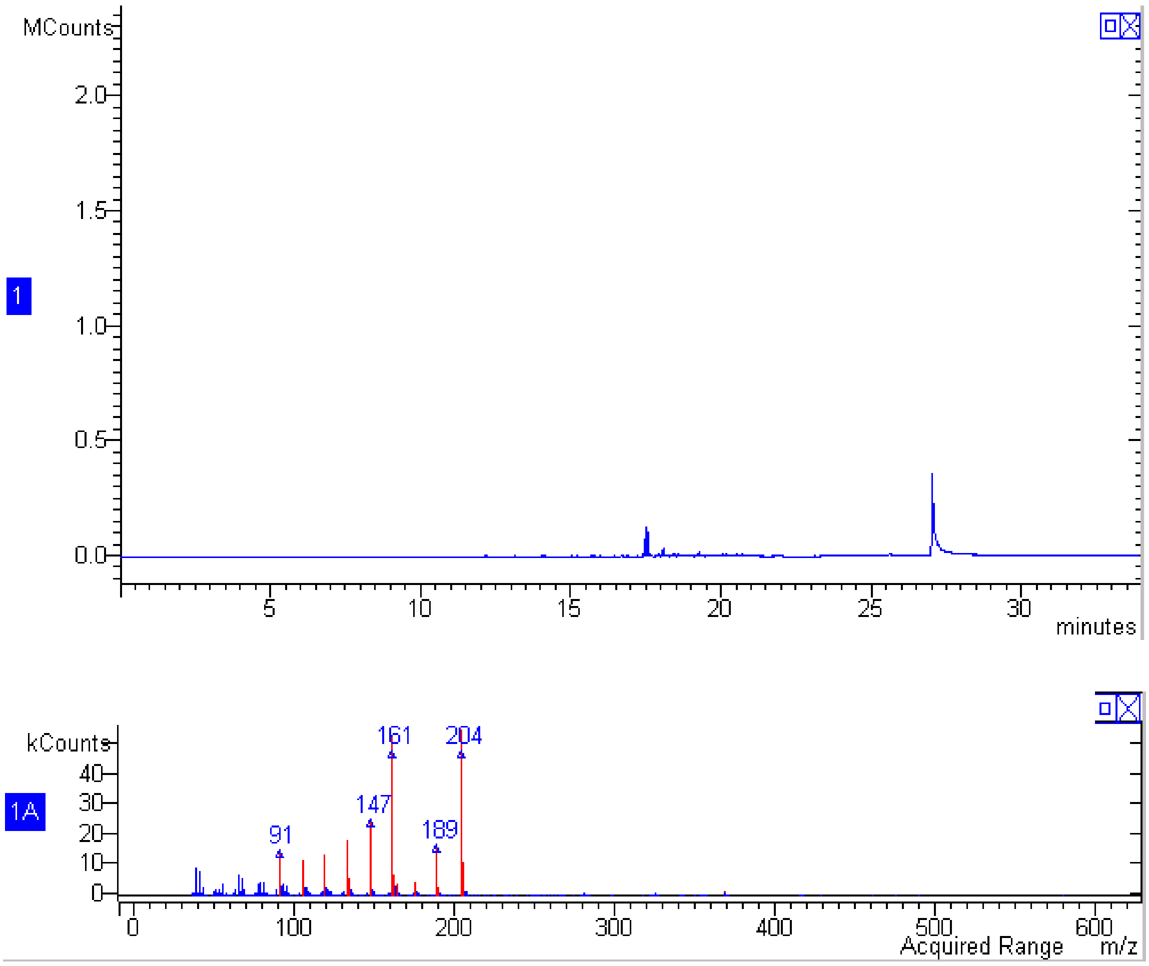

Supplementary data 5: Mass spectra and retention time of 6-epi-ß-verbesinol cumarate in the ethanolic extract
